# Microbiological Safety and the Management of Microbial Resources in Artisanal Foods and Beverages: The Need for a Transdisciplinary Assessment to Conciliate Actual Trends and Risks Avoidance

**DOI:** 10.3390/microorganisms8020306

**Published:** 2020-02-22

**Authors:** Vittorio Capozzi, Mariagiovanna Fragasso, Pasquale Russo

**Affiliations:** 1 Institute of Sciences of Food Production, National Research Council (CNR), c/o CS-DAT, Via Michele Protano, 71121 Foggia, Italy; 2 Department of the Sciences of Agriculture, Food and Environment, University of Foggia, Via Napoli 25, 71122 Foggia, Italy; mariagiovanna.fragasso@gmail.com (M.F.); pasquale.russo@unifg.it (P.R.)

**Keywords:** artisanal fermented food, fermentation, artisanal fermented beverage, safety, risks, spontaneous fermentation, starter cultures, spoilage microbes, pathogens, toxins

## Abstract

Current social and environmental trends explain the rising popularity of artisanal fermented foods and beverages. In contrast with their marketing success, several studies underline a lack of regulations necessary to claim differences occurred from the farm to the fork and to certify high quality and safety standards. Microbial-based fermentative processes represent the crucial phase in the production of fermented foods and beverages. Nevertheless, what are the effects of the application of the “artisanal” category to the management of food fermentations? This opinion paper is built up on this issue by analyzing microbial aspects, instances of innovation, safety issues, and possible solutions. Evidence indicates: (i) a global curiosity to exploit food fermentations as drivers of innovation in artisanal contexts and (ii) an increasing interest of the artisanal producers into management of fermentation that relies on native microbial consortia. Unfortunately, this kind of revamp of “artisanal food microbiology,” rather than re-establishing artisanal content, can restore the scarce hygienic conditions that characterized underdeveloped food systems. We highlight that in the scientific literature, it is possible to underline existing approaches that, surpassing the dichotomy between relying on spontaneous fermentation and the use of commercial starter cultures, depict a “third way” to conjugate interest in enhancing the artisanal attributes with the need for correct management of microbial-related risks in the final products.

## 1. Introduction

Fermentation of edible matrices has been a precious form of food preservation and functional/nutritional enhancement for millennia [1]. The fermentation of edible matrices is the microbial-based process mainly characterized by the conversion of sugars into alcohols and acids, always associated with a subset of secondary microbial-based modifications. On the whole, this so-called “oldest biotechnology” modifies the original matrices into a new food product (e.g., kefir, beer, kombucha, sauerkraut, cheese, miso, kimchi, tempeh, natto, sourdough bread) [2]. In fact, fermented foods or beverages are “produced through controlled microbial growth, and the conversion of food components through enzymatic action” [1]. The fact that this ancient form of preservation was present in human societies worldwide well explains the variety and the diversity of existing fermented foods and beverages [3]. Before the advent of modern microbiology, food fermentations (unconsciously) relied on naturally occurring microorganisms [4]. For example, in the field of sourdough technology by applying the back-slopping, in other terms, “the inoculation of the raw material with a small amount of dough from a previous successful fermentation” [5], while in other production sectors, the practice found different declinations, named, for example, “inoculum enrichment” (general), “*sieroinnesto*” (cheese sector), or “*pied de cuve*” (wine sector) [6]. With the development of tailored microbiological techniques and the rise of industrial food production, to ensure the standardization of consistency, safety, and quality of the final fermented products the scientific community developed the technology of starter cultures: “a microbial preparation of large numbers of cells of at least one microorganism to be added to a raw material to produce a fermented food by accelerating and steering its fermentation process” [7]. As more recently defined, “commercial starter cultures are standardized inoculum to be used for the production of fermented foods. Starter cultures are produced by specialized manufacturers. Rigorous quality assurance and quality control are conducted to ensure the performance, composition, and safety of the culture” [8].

## 2. Globally Fermented Foods and Beverages and the Microbial Diversity Associated with

With a few exceptions (e.g., [9]), all globally fermented foods and beverages are based on ancient artisanal/typical/traditional productions [10]. In order to provide an overview of micro-biodiversity associated to the different edible matrices subjected to the fermentative process, we refer to the nine categories proposed by Tamang et al. [3] that differentiated global products in: i) fermented cereals; ii) fermented vegetables and bamboo shoots; iii) fermented legumes; iv) fermented roots/tubers; v) fermented milk products; vi) fermented and preserved meat products; vii) fermented, dried, and smoked fish products; viii) miscellaneous fermented products; and ix) alcoholic fermented beverages. In Table 1, you can find a list of some of the genera/species of prokaryotic and eukaryotic microorganisms found in association with the fermentative processes in the corresponding categories.

The objective of this list is to provide a brief representation of the dimension of the microbial diversity we deal with in the management of the heterogeneous class of globally fermented foods and beverages. An issue that represents the “living matter” is the basis of the subject of this opinion paper.

## 3. Artisanal Foods and Similar Categories: The Borders and Overlapping Concept

Different terms can be used to describe the degree of originality of a given production: territorially, tipicity, traditionality, communality, and artisanality. They represent a sort of grey scale so blended that we can often confuse them. “Territoriality” measures “the degree of physical connection between a food product and its place of origin,” including the different phases associated with the productive chain, comprising also distribution and final consumption [13]. “Typicity” describes “the physical aspects that distinguish the production process and the final product in as far as they are unique or logically linked to the place of origin” [13]. “Traditionality” measures the originality in terms of “time” since “first appeared in the place of origin.” [13]. “Communality” identifies “the sharing of experience (know-how as well as social) […] by the supply chain actors,” thus quantifying “the degree of horizontal and vertical collaboration among these actors” [13]. “Artisanality” describes processed products “that are made and sold by individual small-scale non-agricultural food producers (bakers, butchers, brewers, etc.) and named after the place (area or town) where the producers are located” [13]. In addition, in order to consider this little glossary exhaustive, we have to include a wording recognized by international intellectual property law, “Geographical Indications” (GIs), whose significance is connected to trademarks. In fact, “Geographical Indication is a sign used on goods that have a specific geographical origin and possess qualities, reputation or characteristics that are essentially attributable to that place of origin” [14].

It appears clear that while the words “territorially,” “tipicity,” and “traditionality” describe/measure different aspects of the connection with a given place of origin, “communality” and “artisanality” are more related to a way to produce, and hence can partially overlap with the previous concepts (i.e., territorially, tipicity, and traditionality). Moreover, we have to underline that, even if artisanal products have become more and more popular in recent years (a popularity also addressable to the increasing interest in social and environmental issues considered linked to artisanal foods), there are a lack of regulations/standards necessary to claim/certify differences occurred in pre-production, production, and post-production that can be of help in recognized artisanal products (particularly differentiating them from the commercial ones) [15]. Taken together, these observations indicate how vague the use of the term artisanal can be and how wide the discretionary margins of the producers remain [15].

## 4. The “Artisanal” Concept Extended to Food Fermentations: Existing Instances of the Stakeholders

The fact that “artisanality” is deeply related to a way to produce foods fosters the idea that all the phases of production can be “handcrafted,” and hence at an individual/small-scale level. Probably, the fermentative process can be considered the crucial phase in the production of fermented foods and beverages. What are the effects of the application of the “artisanal” category to the management of food fermentations? An issue difficult to cope with by reason of the atomistic nature of the artisanal markets of fermented products. However, it is possible to intercept some echoes from the “real world.” In other terms, it is possible to underline some authoritative “voices” susceptible to be considered representative of the existing instances of the stakeholders. Here, we selected two examples in order to testify on the actual trends. An interesting point of view arises listening to Piero Sardo, president of the Slow Food Foundation for Biodiversity Onlus: “With industrial starters, cheese made in the mountains will be only slightly different from cheese made in the plains. […] With the industrial packets, the music is already written and the cheesemaker just plays it” [6]. Hence, one of the more important proponents of Slow Food, an association that builds an international movement around the valorization of artisanally produced foods [16], claimed the importance of avoiding the use of starter cultures in order to restore the artisanal “fermentative” practices. The second example, in the field of gastronomic sciences, deals with a recent “debate” in the journal *Nature Microbiology* on the topic of “artisanal food microbiology” [17,18]. On the one side, the young scientist Arielle Johnson, in a “feature paper,” underlines the relevance of microbial resources as a “culinary tool” to allow “both professional and amateur cooks to create entirely new flavours from existing ingredients” [17]. In other terms, she proposed microorganisms as creative agents in the hands of food-makers and of gastronomic actors. On the other side, Cocolin et al. [18], referring to Johnson’s argumentation, addressed a published letter to the editor of the journal highlighting the interest in the subject, but overall stressing how crucial it can be to “take into adequate consideration the risks of fermenting in non-specialized facilities, such as restaurant kitchens.”

On the whole, these two examples warn about relevant trends in the field of the management of food fermentations at the artisanal level: the renovated interest of artisanal producers into spontaneous fermentation (including “inoculum enrichment” managements that rely on native microbial consortia) and the global interest to exploit food fermentations as drivers of innovation in artisanal contexts. In addition, we have to always remember that “much smallscale fermentation in developing countries is still conducted” exploiting spontaneous microbial consortia [19].

## 5. “Artisanal Fermentation” and the Safety Issues: Concrete Microbial-Related Risks

Here, we propose an overview of the concrete chemical and biological risks associated with microorganisms and to their metabolic products in the context of spontaneous fermentations (a category that includes, as said, inoculum enrichment managements based on indigenous microbial consortia) [20], a way to remember and underline how certain “artisanal fermentations” can pose serious hazards to human health. On the one side, there are the biological contaminants, in other words, the microbial foodborne pathogens found in association with principal categories of fermented foods and beverages (e.g., wine, fermented cereals, cheese, sausages, fermented fish). On the other side, there are the chemical contaminants that are toxic by-products of microbial origin. Pathogenic microbes include *Bacillus cereus*, *Escherichia coli*, *Salmonella* sp., *Escherichia coli* O157:H7, *Listeria monocytogenes*, *Aeromonas*, *Klebsiella*, *Campylobacter*, and *Shigella* sp. and undesirable microbes like *Bacillus cereus*, *Proteus mirabilis*, *Staphylococcus aureus*, *Proteus penneri*, *Enterococcus faecalis*, and *Staphylococcus saprophyticus* [21,22,23]. In particular, fermented products based on animal matrices are extremely vulnerable to contamination [21,24]. Chemical contaminants include several classes of microbial-related molecules including mycotoxins (e.g., aflatoxins, deoxynivalenol, fumonisins, nivalenol, ochratoxin, zearalenone), biogenic amines (cadaverine, histamine, isoamylamine, phenylethylamine, putrescine, and tyramine), ethyl carbamate, and cyanogenic glycosides [21,25,26]. Coming back to microbial-associated hazards for human health, it is essential to separate (i) risks associated with microbial genera/species always undesired in association with fermented matrices, and (ii) genera/species detected in the monitoring of spontaneous fermentation. The first is the case of pathogens, while to the second class often belong producers of mycotoxins, ethyl carbamate, and biogenic amines. Hence, the second class is more insidious, considering that in the same species we can also find strains of protechnological interest and strains liable to produce toxic compounds.

If we focus on the inoculum enrichment managements, one other way to examine the question of microbial-related risks associated with spontaneous fermentations is to consider the positive list of microbial species admitted under the umbrella of status such as Qualified Presumption of Safety (QPS). This list has been developed, and continuously updated, by the European Food Safety Authority (EFSA) to evaluate the safety of biological agents intentionally added during food processing, with the aim of harmonizing the risk management associated with microbial food cultures in Europe [6]. Having a look at the list of microbial species and genera reported in Table 1, it is easy to observe an asymmetry in comparison with the list of QPS microbes. This variance provides us an idea of how difficult is to rely on spontaneous microbial consortia to drive food fermentations and at the same time assure food safety [20].

## 6. The Need for a Transdisciplinary Assessment: Towards a “Third Way” to Conciliate Actual Trends and Risks Avoidance

Health benefits associated with the consumption of fermented foods and beverages are often claimed [1,2,27]. However, this good reputation in terms of nutritional and functional quality represents one more reason to assure a risk management tailored to minimize the probability of contaminations. Consistent microbial-related safety issues deal with the rising interest of the artisanal food sector in spontaneous fermentations (including all the kinds of practices that rely on native microbial communities without the required microbiological and chemical controls). A revamp of “artisanal food microbiology” could not only reestablish the artisanal content, but also restore the scarce hygienic conditions that characterized underdeveloped food systems.

In the scientific literature, it is possible to underline existing approaches that, surpassing the dichotomy between relying on spontaneous fermentation and the use of commercial starter cultures, depict a “third way” to conjugate the interest in enhancing the artisanal attributes by means of tailor-made fermentation strategies with the need of a correct management of microbial-related risks in the final products. In this heterogeneous set of approaches, three main categories of studies can be identified. Firstly, the works that, starting from a genotypic and technological characterization of microbial diversity associated to giving food spontaneous fermentation, proposed the design of a multi-strains starter culture to be used in the associated food production in order to improve the “unique qualities” recognized as artisanal attributes [4,6,20]. In addition, there are numerous examples of biotechnological and analytical methodologies that allow the monitoring of the microbial-based contaminants associated with a given spontaneous food fermentation [28,29,30,31]. Finally, the case of studies that applied the safety standard used to assess the compliance of single microorganisms in order to evaluate the safety of the microbial diversity at the basis of inoculum enrichment management [32]. However, neither of these approaches proposed represent reference solutions for the desired renaissance in artisanal food microbiology. The reason why is probably connected i) with the general, limited education in microbiology of the key actors working in the field [33] and ii) with the poor coordination among the approaches belonging to a different disciplinary environment and among quadruple helix stakeholders [34]. Many questions remain open when dealing with different sectors, e.g., regulatory compliance, right importance in product specifications, low-cost approach to produce microbial biomass, and potentiating comprehension and approval of the consumers and of the stakeholders, even if, concerning specific aspects, some resolutions have been suggested [14,35].

## 7. Conclusions

Fermented foods and beverages represent a worldwide category of edible products with a huge scientific dimension, social relevance, and economic significance. To provide a general idea of the present global interest in this field, we can remember that about one-third of food produced for human consumption worldwide is a fermented edible matrix [36]. Furthermore, the attention of prestigious international institutions, such as the World Health Organization (WHO) and Food and Agriculture Organization (FAO) of the United Nations (UN), to the importance of microbiological risks assessment [37] and of traditional fermented food and beverages [19] contributes to testify to the relevance of the discussed topic. In this opinion paper, we underline the urgency of a holistic effort of all the stakeholders involved to conciliate actual trends and risk avoidance in the management of microbial resources in artisanal fermented foods and beverages by means of a shared global regulatory/applicative framework. A major control of the microbiology related to the fermentation of artisanal foods can represent a milestone in the valorization of the biotechnological potential associated with characterized strains belonging to spontaneous fermentation. Additionally, the valorization of selected microbial resources can be crucial to improve, more generally, the global quality of artisanal fermented products by: (i) enhancing biocontrol activities [38,39,40], (ii) ameliorating nutrient contents [41,42], (iii) augmenting functional properties [43,44], (iv) increasing organoleptic features [45,46], and (v) helping to cope with global issues such as the negative influences of climate changes [47]. Moreover, by reducing the negative risks and improving beneficial effects connected to artisanal fermented products, we might add a further piece to the puzzle of pursuing a One Health approach in the field of fermented foods [48]. Certainly, the recent advances in genomes and metagenomic analysis, allowing innovative safety assessment and fermentative monitoring [22,23,49,50,51,52], could provide new approaches to cope with the needs of artisanal food microbiology.

## Figures and Tables

**Table 1:** A list of microorganisms involved in the fermentative processes of the main categories of globally fermented foods with examples of microorganisms involved in the corresponding fermentative processes. Information reported in accordance to Tamang et al. [3], with integrations from other studies [11,12].

Major Groups of Globally Fermented Foods	Microorganisms Involved in the Fermentation Process
Fermented cereals	*Lactococcus* sp., *Leuc. mesenteroides*, *Lb. delbrueckii*, *Lb. fermenti*, *Lb. coryniformis*, *Leuconostoc* sp., *Ped. acidilactis*, *Ped. cerevisae*, *Streptococcus* sp., *Ent. faecalis*, *Ent. cloacae*, *Weissela* sp., *Bacillus amyloliquefaciens*, *Klebsiella pneumoniae*, *Aerobacter* sp., *Candida cacaoi*, *Cand. fragicola*, *Cand. glabrata*, *Cand. kefyr*, *Cand. pseudotropicalis*, *Cand. sake*, *Cand. tropicalis*, *Debaryomyces hansenii*, *Deb. tamarii*, *Issatchenkia terricola*, *Kluyveromyces marxianus*, *Sacch. cerevisiae*, *Torulopsis candida*, *Tor. holmii*, *Monascus purpureus*, *Rhizopus* sp., *Cephalosporium* sp., *Mucor* sp., *Fusarium* sp., *Penicillum* sp., *Aspergillus* sp., *Endomycopsis* sp., *Hansenula* sp.
Fermented vegetables and bamboo shoots	*Leuc. mesenteroides*, *Leuc. citreum*, *Leuc. gasicomitatum*, *Leuc. fallax*, *Leuc. kimchii*, *Leuc. inhae*, *W. koreensis*, *W. kimchii*, *W. cibaria*, *Lb. plantarum*, *Lb. sakei*, *Lb. delbrueckii*, *Lb. buchneri*, *Lb. brevis*, *Lb. fermentum*, *Ped. acidilactici*, *Ped. pentosaceus*, *Lc. lactis*, *Ent. durans*, *Tetragenococcus halophilus*, *Bacillus subtilis*, *B. lichniformis*, *B. coagulans*, *B. cereus*, *B. circulans*, *B. firmus*, *B. pumilus*, *B. sphaericus Candida* sp., *Halococcus* sp., *Haloterrigena* sp., *Kluyveromyces* sp., *Lodderomyces* sp., *Natrialba* sp., *Natronococcus* sp., *Pichia* sp., *Saccharomyces* sp., *Sporisorium* sp., *Trichosporon* sp., *Pseudomonas* sp., *Halorubrum orientalis*, *Halosarcina pallid*, *Sphingobium* sp., *Thalassomonas agarivorans*
Fermented legumes	*Bacillus subtilis*, *B. brevis*, *B. circulans*, *B. coagulans*, *B. licheniformis*, *B. pumilus*, *B. sphaericus*, *Lysinibacillus fusiformis Rhiz. oligisporus*, *Rhiz. arrhizus*, *Rhiz. oryzae*, *Rhiz. stolonifer*, *Asp.niger*, *Citrobacter freundii*, *Enterobacter cloacae*, *K. pneumoniae*, *K. pneumoniae* subsp. *ozaenae*, *Pseudomas fluorescens*, *Lb. fermentum*, *Lb. lactis*, *Lb. plantarum*, *Lb. reuteri*, *Pantoea agglomerans*, *P. gaananatis*, *Enterococcus* sp., *Pseudomonas* sp., *Rhodococcus* sp., *Asp.* *oryzae*, *Asp.* *flavus*, *Asp.* *fumigatus*, *Asp.* *niger*, *Asp.* *retricus*, *Asp.* *spinosa*, *Asp.* *terreus*, *Asp.* *wentii*, *Botrytis cineara*, *Ped. halophilus*, *Staphylococcus* sp.
Fermented roots/tubers	*Bacillus* sp., *Lb. plantarum*, *Leuc. mesenteroides*, *Lb. cellobiosus*, *Lb. brevis; Lb. coprophilus*, *Lc. lactis; Leuc. lactis*, *Lb. bulgaricus*, *Klebsiella* sp., *Leuconostoc* sp., *Corynebacterium* sp., *Candida* sp., *Micrococcus* sp., *Pseudomonas* sp., *Acinetobacter* sp., *Moraxella* sp., *Rhizopus* sp.
Fermented milk products	*Lc. lactis* subsp. *cremoris*, *Lc. lactis* subsp. *lactis*, *Lb. alimentarius*, *Lb. biofermentans*, *Lb. brevis*, *Lb. delbrueckii* subsp. *delbrueckii*, *Lb. delbrueckii subsp.* *lactis*, *Lb. farciminis*, *Lb. helveticus*, *Lb. casei*, *Lb. plantarum*, *Lb. salivarius*, *Leuconostoc* spp., *Strep. thermophilus*, *Ent. durans*, *Ent.faecalis*, *Ent. faecium*, *Ped. pentosaceous*, *Ped. acidilactici*, *Bifidobacterium* spp., *Staphylococcus* spp., *Brevibacterium linens*, *Propionibacterium freudenreichii*, *Weissella confusa*, *Candida* sp., *Saccharomycopsis* sp., *Debaryomyces hansenii*, *Geotrichum candidum*, *Penicillium camemberti*, *P. roqueforti*, *Pichia kudriavzevii*
Fermented and preserved meat products	*Lb. pentosus*, *Lb. plantarum*, *Lb. brevis*, *Lb. paracasei*, *Lb. fermentum*, *Lb. acidipiscis*, *Lb. farciminis*, *Lb. rossiae*, *Lb. fuchuensis*, *Lb. namurensis*, *Lc. lactis*, *Lb. sakei*, *Leuc. citreum*, *Leuc. fallax*, *Ped. acidilactici*, *Ped. pentosaceus*, *Ped. stilesii*, *W. cibaria*, *W. paramesenteroides*, *Ent. faecalis*, *Ent. faecium*, *Ent. hirae*, *Bacillus subtilis*, *B. mycoides*, *B. thuringiensis*, *Staphylococcus spp.*, *Micrococcus* sp.
Fermented, dried, and smoked fish products	*Lc. lactis*, *Lb. plantarum*, *Lb. pobuzihii*, *Lb. fructosus*, *Lb. amylophilus*, *Lb. coryniformis*, *Ent. faecium*, *Ent. faecalis*, *Bacillus subtilis*, *B. pumilus*, *B indicus*, *Micrococcus* sp., *Staphylococcus cohnii* subsp. *cohnii*, *S. carnosus*, *Strep. faecalis*, *Sarcina* sp., *Corynebacterium* sp., *Tetragenococcus halophilus* subsp. *flandriensis*, *Pseudomonas* sp., *Halococcus* sp., *Halobacterium salinarium*, *H. cutirubrum*, *Clostridium irregular*, *Azorhizobium caulinodans*, *Candida* sp., *Saccharomycopsis* sp.
Miscellaneous fermented products	*Acetobacter aceti* subsp. *aceti*, *Acetobacter pasteurianus*, *Acetobacter polyxygenes*, *Acetobacter xylinum*, *Acetobacter malorum*, *Acetobacter pomorum*, *Candida lactis-condensi*, *Candida stellata*, *Hanseniaspora valbyensis*, *Hanseniaspora osmophila*, *Saccharomycodes ludwigii*, *Sacch*. *cerevisiae*, *Zygosaccharomyces bailii*, *Zygosaccharomyces bisporus*, *Zygosaccharomyces lentus*, *Zygosaccharomyces mellis*, *Zygosaccharomyces Pseudorouxii*, *Zygosaccharomyces rouxii*
Alcoholic beverages	*Saccharomyces cerevisiae*, *Candida colliculosa*, *C. stellata*, *Hanseniaspora uvarum*, *Kloeckera apiculata*, *Kl. thermotolerans*, *Torulaspora delbrueckii*, *Metschnikowia pulcherrima*, *Pichia fermentans*, *Schizosaccharomyces pombe*, *Hanseniaspora uvarum*, *Oenococcus oeni*, *Lb. plantatum*

*Ent.*, *Enterococcus*; *Lb.*, *Lactobacillus*; *Lc.*, *Lactococcus*; *Leuc.*, *Leuconostoc*; *W.*, *Weissella*; *Ped.*, *Pediococcus*.
